# Rotation Disk Process to Assess the Influence of Metals and Voltage on the Growth of Biofilm

**DOI:** 10.3390/ma9070568

**Published:** 2016-07-12

**Authors:** Dana M. Barry, Paul B. McGrath

**Affiliations:** 1 Departments of Chemical & Biomolecular Engineering, Clarkson University, Potsdam, NY 13699, USA; 2 Department of Electrical and Computer Engineering, Clarkson University, Potsdam, NY 13699, USA; mcgrath@clarkson.edu; 3 Center for Advanced Materials Processing (CAMP), Clarkson University, Potsdam, NY 13699, USA

**Keywords:** rotation disk reactor, biofilm, bacteria, latex, metals, voltage

## Abstract

Biofilms consist of not only bacteria but also extracellular polymer substrates (EPS). They are groups of microorganisms that adhere to each other on a surface, especially as a result of exposure to water and bacteria. They can pose health risks to humans as they grow in hospital settings that include medical supplies and devices. In a previous study, the researchers discovered that bacteria/biofilm grew well on wetted external latex, male catheters. These results concerned the investigators and encouraged them to find ways for prohibiting the growth of bacteria/biofilm on the male catheters (which are made of natural rubber). They carried out a new study to assess the influence of metals and voltage for the growth of bacteria on these latex samples. For this purpose, a unique Rotation Disk Reactor was used to accelerate biofilm formation on external male catheter samples. This setup included a dip tank containing water and a rotating wheel with the attached latex samples (some of which had single electrodes while others had paired electrodes with applied voltage). The process allowed the samples to become wetted and also exposed them to microorganisms in the ambient air during each revolution of the wheel. The results (as viewed from SEM images) showed that when compared to the control sample, the presence of metals (brass, stainless steel, and silver) was generally effective in preventing bacterial growth. Also the use of voltage (9.5 volt battery) essentially eliminated the appearance of rod shaped bacteria in some of the samples. It can be concluded that the presence of metals significantly reduced bacterial growth on latex and the application of voltage was able to essentially eliminate bacteria, providing appropriate electrode combinations were used.

## 1. Introduction

Biofilms can be difficult to define as they have different structures and compositions depending upon their environment. They contain various components such as microbial cells, polysaccharides, proteins, and water channels to allow for the delivery of nutrients and the removal of waste products [1]. However, the main component is water which can be up to 95% of their wet weight [2]. Biofilms grow by recruiting microorganisms and by cell division. They use Quorum Sensing (QS) to regulate colonization, where microbial cell-to-cell communications turn on certain bacterial activities [3]. Most bacteria that cause health problems exist in biofilms [4]. As they are embedded within a self-produced EPS (extracellular polymer substrate), this makes them resistant to antibiotics. Biofilms can grow on different surfaces in a wide variety of environments. They are found on floors, counter tops, food, slimy rocks, and in dental plaque. They are also present in hospital settings that include an array of medical supplies and devices [5,6]. Therefore, from a medical point of view, in prior work, the researchers carried out a preliminary investigation to assess the biofilm forming ability of a variety of items used in hospital settings [7]. They designed and used a unique Rotation Disk Reactor for their study. (This reactor will be described in the Results and Discussion section). The reactor was created not only to simulate a hospital setting (where items are exposed to moisture and air), but to accelerate biofilm growth without resorting to artificial acceleration substrates. This setup included a dip tank with deionized/distilled water and a rotating wheel that could hold multiple samples. In this prior work, the process allowed the samples to become wetted and exposed to microorganisms in the ambient air during each revolution of the wheel. The six medical items tested were the nebulizer (made of polyvinyl chloride), syringe (made of polypropylene), pipette (made of polyethylene), cannula tubing (made of polyvinyl chloride), a suction catheter (made of polyvinyl chloride with added plasticizers), and the external male catheter (made of latex, natural rubber). After several weeks of testing, the latex catheter sample was wetted and had a surface coating that looked white and slimy. The other samples were still hydrophobic. Upon further testing, SEM images for the latex catheter samples displayed rod shaped bacteria.

The rapid growth of bacteria on the latex catheter samples concerned the researchers. They wanted to carry out a separate study to find methods of inhibiting bacteria/biofilm growth on latex, especially since it is used in catheters and other medical devices. They read and considered journal articles about latex and biofilm [8,9,10,11,12]. However, that information did not directly relate to their work, which they considered to be a new area of study. After finding examples in the literature where metal ions and the use of electric fields exerted antibacterial effects, the investigators launched their second preliminary study [13,14,15,16,17]. This time they assessed the influence of metals and voltage for the growth of biofilm on latex samples.

Once again, the unique Rotation Disk Reactor and setup conditions (exposure to ambient air and wetted with deionized/distilled water) were used for three trials of the experiment. All of the samples were latex from the external male catheter. For each trial, the test items included a control sample of latex, latex with silver, latex with stainless steel, latex with brass, latex with both stainless steel and brass, and latex with paired electrodes and applied voltage. This work is important from a medical point of view because its goal was to prohibit the growth of bacteria/biofilm on latex that is used in catheters and other clinical devices.

## 2. Results and Discussion

Initially, all samples were hydrophobic. After five days of operation they began to display areas of light discoloration which may be associated with the loss of hydrophobicity. The samples were all tubular in shape and had openings facing outward that were seen to hold water throughout the revolution of the wheel. The tip and inside opening of each sample were the first to exhibit surface wetting and the attendant discoloration. 

**Figure 1:**
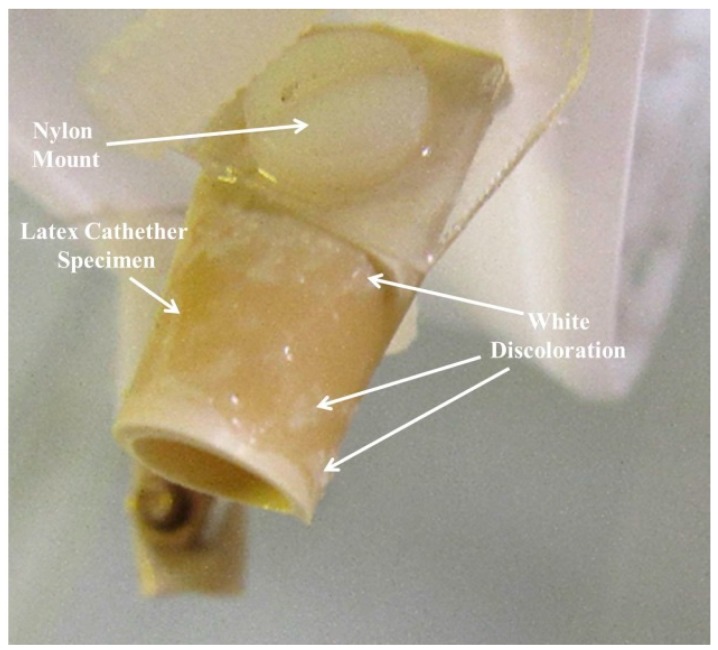
Control sample 0 (from trial #1) shows areas of white discoloration that may be associated with surface wetting.

After the second week of testing (during the three trials), sample 0 (the control), sample 8 (latex with brass as negative & stainless steel as positive electrodes), and sample 7 (latex with brass as positive & stainless steel as negative electrodes) were all at least 80% discolored on the front side. Figure 1, a good representation of the three trials, shows areas of white discoloration for the control sample. This is the type of discoloration that we refer to in our sample observations. During the three trials, the following samples (sample 4: latex with stainless steel & brass; sample 5: latex with stainless steel & stainless steel; sample 6: latex with brass & brass; sample 7: latex with brass positive & stainless steel negative electrodes; and sample 8: latex with brass as negative & stainless steel as positive electrodes) were at least 75% discolored on the backside. Sample 1 (latex with silver), sample 2 (latex with stainless steel) and sample 3 (latex with brass) were 60% or less discolored on both their front and back sides. The least discolored sample of all was sample 2 (latex with stainless steel). It was about 30% discolored on the front and about 15% discolored on the back.

**Figure 2:**
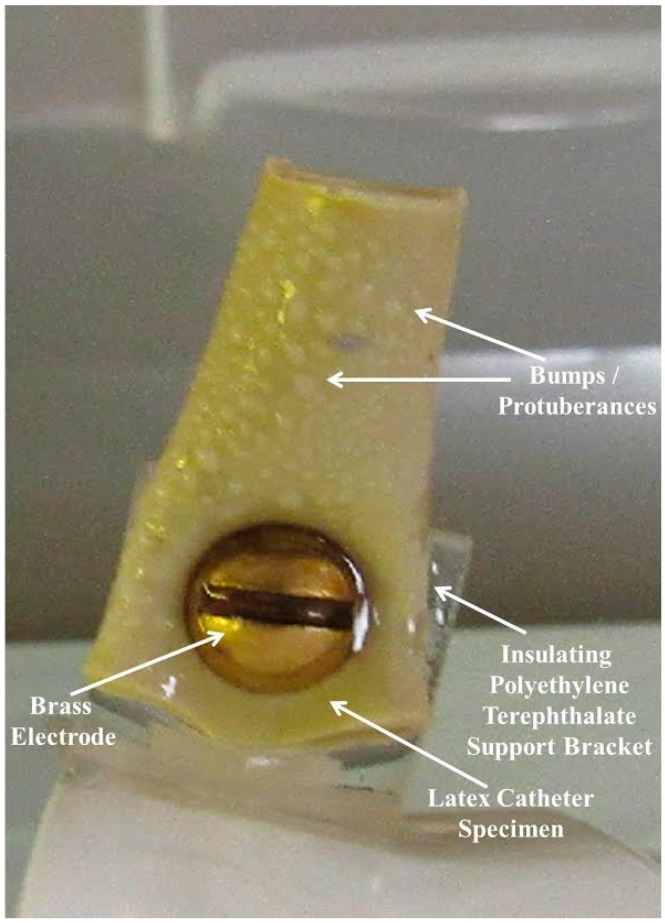
Sample 3 (from trial #1) with brass electrode, showing bumps/protuberances on its front face.

After three weeks of testing, all samples appeared to be hydrophilic and to contain bumps/protuberances of various sizes and amounts. See Figure 2. It displays bumps on the front face of sample 3 (latex with brass), which was typical for the three trials. These are the types of bumps that we refer to in our sample observations. Refer to Table 1 for a summary of the overall weekly observations (for the three trials) during the testing period.

The protuberances on the sample surfaces appear to have been created when the solvent accumulated under the surface. This accumulation suggests that deterioration was occurring. The latex catheter samples were made of natural rubber, which is mostly a polymer of isoprene units [18]. This uncured form of rubber has limited strength and deteriorates over time. For example, large rubber molecules can break up as they oxidize in air due to oxygen molecules attacking the double bonds [19,20]. The formation of protrusions on the samples indicated a change and growing weakness in the material. It is interesting to note that during the three experiments, sample 7 (brass as positive electrode and stainless steel as negative electrode) and sample 8 (brass as negative electrode and stainless steel as positive electrode) were wet and smooth and essentially free of the degraded surface. They had very few bumps. 

### 2.1. SEM Data

**Figure 3:**
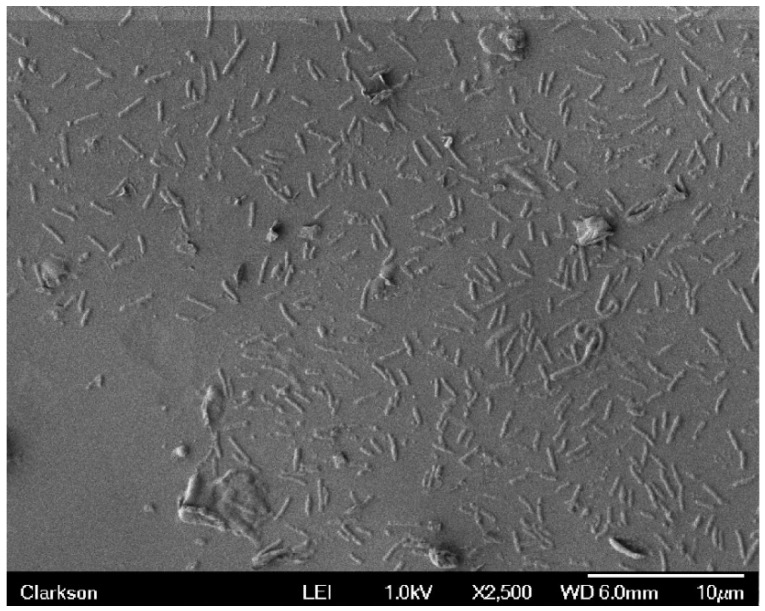
The SEM image (at 2500×) of the aged control latex sample displays many rod shaped bacteria.

The SEM images and specific data displayed in this section are from experimental trial #1. However, it should be mentioned that the data obtained in experimental trials #2 and #3 did not vary much from that of experimental trial #1. Therefore, the results of experimental trial #1 are a good representation of the overall research project. These results are provided. The control sample, sample code 0, exhibited significant bacterial growth as seen in Figure 3. In this figure the bacteria count was 494. Sample 1, with a single silver electrode, had 20 bacteria in the same SEM viewing area. The sample with the single stainless steel electrode, sample 2, showed 12 bacteria in a film, while sample 3, using a single brass electrode revealed 7 bacteria for the same viewing area. NOTE: All sections of each sample were scanned. Then a bacteria count was made for the highest populated area on the surface of each sample. It can be seen that the presence of a metallic electrode was very effective in preventing the growth of bacteria on the latex substrate in its immediate vicinity. The electrode with the greatest influence was brass followed by stainless steel and then silver. 

Metal ions, including copper, zinc, and silver, have been shown to be toxic to bacteria by the oligodynamic effect. Studies suggest that copper alters the structure of proteins or destroys them so they can no longer perform the required functions to keep bacteria active [21]. The actual mechanisms for the various metal ion toxic effects are complex and still under investigation. However, there have been some recently discovered processes involving silver. Silver interrupts the ability of a bacterial cell to form the chemical bonds that are necessary for survival [22]. These bonds are needed to produce the physical structure of the cell. Iron ions generally benefit the production of biofilm [23]. However, the opposite effect was observed in this study. This contradiction could be attributed to the presence of ions from chrome and nickel (both of which were present in the surface film of the stainless steel). These ions could show antibacterial and antifouling effects. 

**Figure 4:**
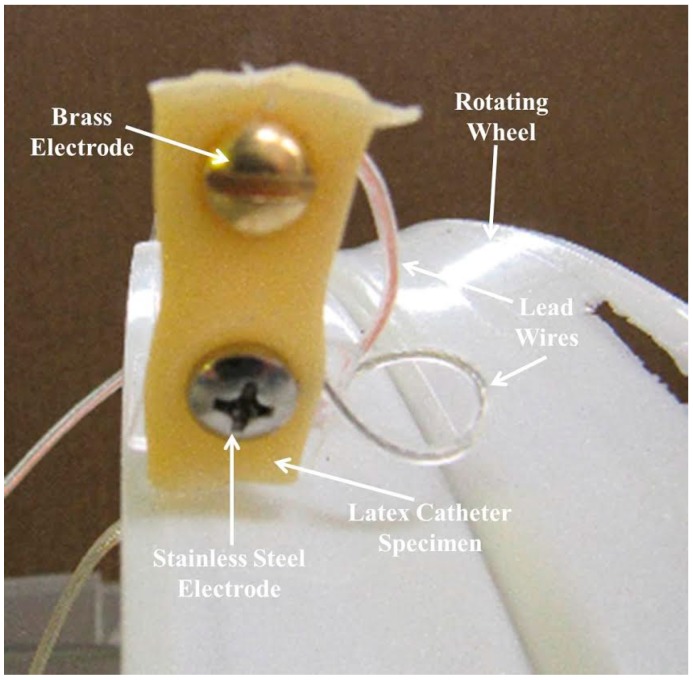
Sample 7, latex with electrically biased stainless steel and brass electrodes at the start of the experiment.

Effects of paired electrode configurations were obtained by examining the latex sections taken from the front face of these samples. An example of this configuration is shown for sample 7 in Figure 4. This sample represents latex with electrically biased stainless steel and brass electrodes, where the brass is positive and the stainless steel is negative. This photo was taken just before the experiment commenced, so it shows the original appearance of the latex catheter samples (starting materials). 

**Figure 5:**
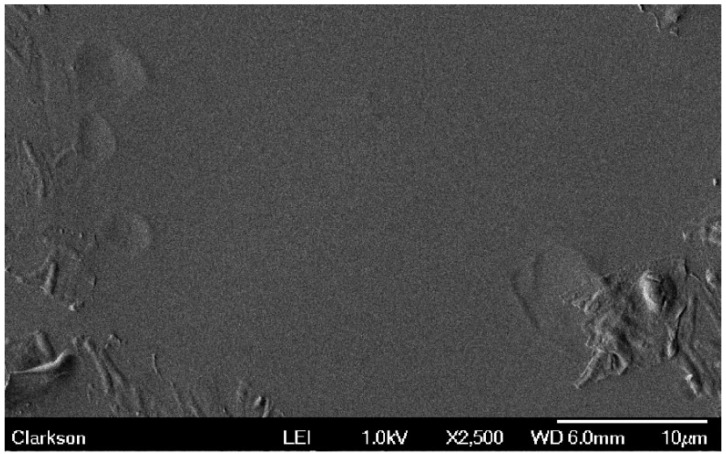
The SEM image (at 2500×) of the aged Sample 5 (stainless steel and stainless steel with added voltage) is essentially free of rod shaped bacteria. The rough edges show aged portions of the sample.

When stainless steel and brass (sample 4) were used together, without external electrical bias, 24 bacteria were observed on the latex substrate in the SEM image area for trial #1, with similar results for trial #2 and trial #3. Images were also obtained for the four samples that had external voltages applied. These used a variety of stainless steel and brass electrode combinations. Sample 5 had two stainless steel electrodes while sample 6 had two brass electrodes. These electrode combinations resulted in essentially no bacteria being observed on their respective latex substrates for all three trials. Figure 5 shows a representative SEM image for sample 5 (two stainless steel electrodes).

**Figure 6:**
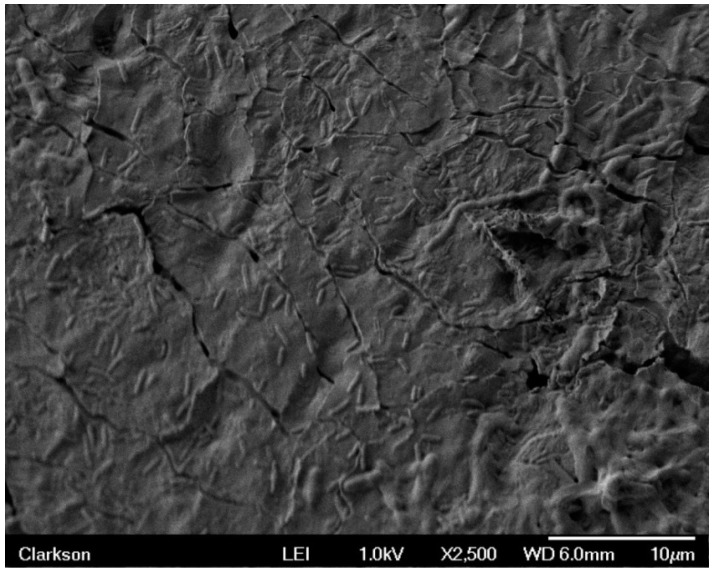
Sample 7 showing surface texture and separation of biofilm with visible bacteria.

**Figure 7:**
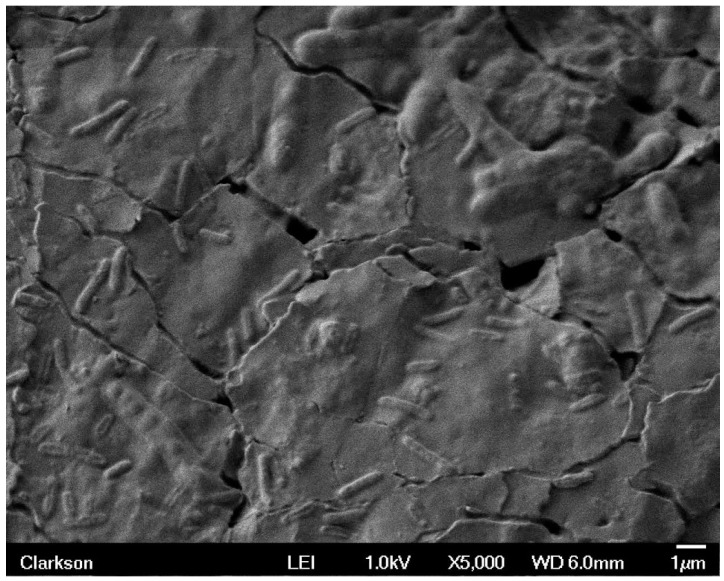
This image has a magnification of 5000× and provides a more detailed image than Figure 6 of the biofilm and bacteria. In this image, it can be seen that separation from the latex substrate has occurred.

Sample 7 had stainless steel and brass electrodes with dc bias such that the brass electrode was positive and the stainless steel negative, while sample 8 had stainless steel and brass electrodes with the opposite bias applied. The SEM images revealed very few bacteria for sample 8 in all three trials. However, about 273 were present in sample 7 during trial #1. Sample 7 is displayed in Figure 6 and Figure 7. Figure 6 shows surface texture and a separation of biofilm with visible rod shaped bacteria. Figure 7 has magnification of 5000× and provides a more detailed image of the biofilm. In this image, it can be seen that separation from the latex substrate has occurred. This disruption of the film most likely occurred during drying and preparation for SEM imaging. In addition to discrete sections of biofilm, individual bacilli can be seen lying close to the surface.

From the bacterial counts obtained for samples 4, 7, and 8, there appears to be an interaction between electrode materials and the external applied electric field. For sample 4, stainless steel and brass without external bias, there was a significant reduction in observed bacteria when compared to the control. As the section examined was taken mid-way between the electrodes and not from the immediate vicinity of either metal, the result for the unbiased case can be considered to be related to the electrochemical potential difference of the two metals rather than the metals themselves. An open circuit voltage of 100 mV was measured between the two electrodes while they were immersed in the aging solution. The polarity was such that the stainless steel was positive and the brass negative. This is consistent with the observation that stainless steel is more positive than brass in the electrochemical potential series for commonly found electrolytes. Under this condition, it is likely that Cu^2+^ and zinc ions are released by the brass electrode as it undergoes galvanic corrosion [24]. For sample 8, there were areas of metallic deposits that can be attributed to electrolytic action from the applied electrical bias. The presence of the deposited ions can be seen to strongly inhibit bacterial growth. No such deposits were seen for sample 7, where the bias appears to have negated the antibacterial tendency of the unbiased electrode combination.

It should be mentioned that the 9.5 V dc is high enough to generate hydrogen gas and oxygen gas from the electrode. Reactions could take place. Chemical corrosion can be seen as oxidation of metals, which occurs by the action of dry gases [25,26]. 

Application of electric fields and the associated currents have been shown to exert antibacterial effects [27]. They are believed to influence the orientation of membrane proteins and the metabolic and developmental processes of bacterial cells. It has been suggested that these antibacterial activities result from the toxic substances produced as a result of electrolysis, oxidation of enzymes, and possibly membrane damage with leakage of the necessary cytoplasmic constituents [28].

### 2.2. Rotation Disk Reactor Compared to Others

For this study, a unique Rotation Disk Process was used to accelerate the surface wetting of samples and biofilm formation. While various types of biofilm reactors exist, each has its own use and limitations. A bioreactor is generally a system where chemical processes, that involve organisms or active substances derived from them, take place. In industry, these processes are often carried out in vessels made of stainless steel and the reactions may be aerobic or anaerobic. 

A few types of bioreactors are presented and described. Tubular reactors can be used to study the effect of biofilm on pressure drop [29]. A tubular reactor consists of vertical or horizontal arranged tubes (generally made of transparent plastic or borosilicate glass), connected together to a pipe system. Constant circulation (flow) is kept up by a pump at the end of the system. The conversion of chemicals is a function of the position within the reactor rather than of time. The horizontal form is used in fermentation and wastewater treatment processes [30].

The packed bed reactor is popular because it imitates flows in nature like those in porous media. For this reactor, cells are immobilized on large particles, which do not move with the liquid. Packing items include ceramics, glass, wood, etc. These reactors often have blockages and poor oxygen transfer [31].

Several bioreactors used in industry include batch bioreactors, photobioreactors, and membrane bioreactors. Batch bioreactors usually contain a tank with an agitator, a heat/cooling system, a feeding pump for adding ingredients, an aeration system, and an effluent attachment. After the reaction is finished, the contents are removed. The reactor is cleaned, refilled with ingredients, and the process starts again [32]. Photobioreactors incorporate a light source and are used to grow organisms such as moss and algae [33]. The membrane bioreactors provide an option (for separating solids and liquids) to the conventional gravity-based method of settling tanks. They combine a membrane for microfiltration or ultrafiltration with suspended growth reactors and are used for municipal and wastewater treatment [34,35,36].

Biofilm reactors generally have three operating configurations. They can have a no recycle format which means that fluent enters the reactor and effluent leaves. Another option is to have a recycle mode and the third is a recycle method that includes a mixing chamber. Researchers in Japan use a continuous flow biofilm reactor that includes recycling and a chamber for mixing in order to test various samples [37]. They pump water from a tank through an acrylic column (that holds samples) and then back to the tank. A fan blows ambient air into the tank so it can mix with the water. This technique is useful for certain purposes. However, it allows a continuous flow of water over the samples throughout the duration of the investigation. 

The intent of this study by Barry and McGrath was to emulate a mixed water and air environment. Medical items in hospitals are not continuously submerged in water; they are more apt to be exposed to both moisture and air. With the unique Rotation Disk Process the samples are cycled, where they are alternately immersed in water followed by an air exposure period. The testing apparatus includes a high density polyethylene (HDPE) dip tank containing 30 L of deionized/distilled water (in order to start the investigation with purified water) and a rotating wheel. The wheel was designed to hold multiple samples and to operate at three revolutions per minute (RPM). This rotation rate was selected in order to both simulate a hospital environment and to accelerate the surface wetting of samples and biofilm formation. 

The wheel was labeled with station numbers 0–8. Each station represented the location of an attached medical item (latex catheter samples with various combinations of electrodes and voltage). The samples were secured to the wheel with nylon hardware for our previous work. For this work the samples were attached to the wheel with the aid of insulating polyethylene terephthalate support brackets. All samples, with the exception of the control, were mounted using metallic electrodes. The attachment design of our reactor allowed the samples to be observed from various positions including top view, bottom view, and side view during testing. A motor with a reduction gear was used to turn the wheel and alternately dip each sample into the water. This continued for 24 h a day throughout the duration of the experiments. The wheel motion through air and water ensured that microorganisms in the ambient air continued to mix with the water in the dip tank. The unique Rotation Disk Reactor is versatile in that it can accommodate a variety of experiments (such as those with or without voltage, etc.). However, it was specifically designed to carry out our research involving bacteria, biofilm, and medical items.

## 3. Materials and Methods 

**Figure 8:**
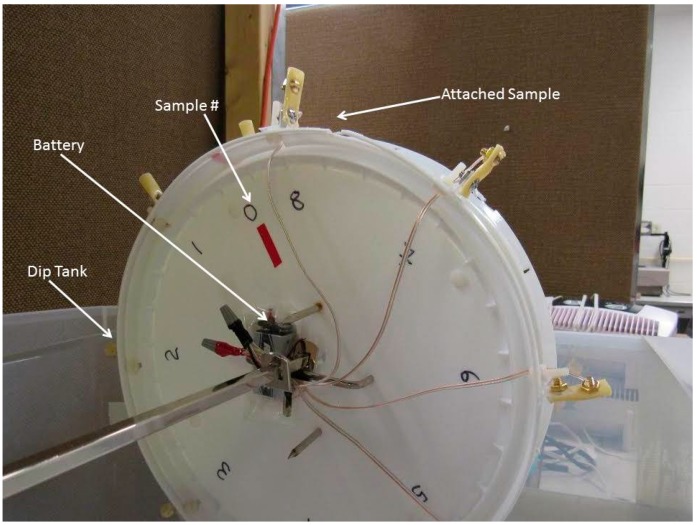
Rotation Disk Reactor with attached samples and dip tank.

A Rotation Disk Process was used for this study, which took place in a laboratory setting. The apparatus included a HDPE dip tank containing 30 L of deionized/distilled water, a rotating wheel on which the samples were mounted, and a motor to turn the wheel at 3 RPM. The rotating disk had sample attachment stations numbered 0–8 as shown in Figure 8. Three experimental trials were carried out using the Rotation Disk Process and the same testing conditions.

The samples were repeatedly immersed in water followed by an air exposure period. The continuous motion of the wheel ensured that microorganisms in the ambient air were afforded the opportunity to mix with the water in the dip tank. As medical items in hospitals are often exposed to both moisture and air, this system provided a comparable environment in an accelerated manner.

The water level in the tank was kept constant and the temperature, conductivity, and pH were continually monitored. Throughout the test, the samples were observed for wettability and biofilm formation. Observations were made using the unaided eye, magnifiers, cameras, and a scanning electron microscope. 

### 3.1. Test Samples

For each experimental trial, nine samples were prepared from commercially manufactured external latex catheters, made of natural rubber. The samples were attached to the wheel with the aid of insulating polyethylene terephthalate support brackets. All samples, with the exception of the control, were mounted using metallic electrodes. Some samples had single electrodes while others had electrode pairs spaced 10 mm apart. For four of the samples with paired electrodes, a 9.5 V dc potential was applied to their terminals for the duration of each test.

Sample NumberElectrode Details0control: nylon mounted1single silver2single stainless steel3single brass4stainless steel—brass5stainless steel—stainless steel with voltage applied6brass—brass with voltage applied7brass positive—stainless steel negative with voltage applied8brass negative—stainless steel positive with voltage applied

### 3.2. Observations

The Rotation Disk Process was used to simultaneously test the nine latex samples for wettability and biofilm formation. Observations were made using the unaided eye, magnifiers, and cameras. Each test was conducted for about a 33 day period. Throughout the entire research project, the average parameters for the water in the tank were: temperature 19.2 °C, pH 5.82, conductivity 0.65 μS/cm. At the end of each test period, a 5 × 5 mm section was cut from the front face of each sample and sputter coated in preparation for microscopy. For the single electrode configurations, the samples for viewing were cut from an area immediately adjacent to the mounting electrode on the front face. For the samples with two electrodes, sections were taken from the center region equidistant from each electrode also on the front face. The prepared sections were viewed using a JEOL JSM-7400F Field Emission SEM (Tokyo, Japan), providing secondary electron imaging (SEI). All sample images were configured with a magnification of 2500 and a voltage of 1 kV. The resulting image provided a SEM viewing area measuring approximately 50 × 40 μm.

## 4. Conclusions

This investigation examined the wettability of samples and the attendant growth of biofilm. The main thrust was to extend the previous study [38] and assess the influence of metals and voltage on the growth of biofilm on latex used in catheters and other medical items. The experiment was carried out in a laboratory setting, using a unique Rotation Disk Process which was designed to more realistically simulate a hospital setting and to accelerate the biofilm formation of the samples. The results showed that the presence of metals was generally effective in preventing bacterial growth. Brass was seen to be the most effective single metal with less than 1% of the surface covered with the rod shaped bacteria. The next best metal was stainless steel where about 1% of the latex substrate was seen to be covered. The least effective metal was silver, which permitted about 5% bacterial coverage. When brass and stainless steel electrodes were used together on a single latex substrate, the region between the electrodes allowed bacteria to exist over 5% of the surface. 

The use of voltage essentially eliminated the appearance of rod shaped bacteria in two of the samples. When like metals were used for the electrodes, stainless steel with stainless steel, and brass with brass, there were essentially no visible bacteria. When different metals were used on the same latex sample, the results were found to be polarity dependent. When brass was used as the negative electrode and stainless steel as the positive electrode, very few bacteria were evident in the SEM images. Bacteria were not noticeable in trial #1 and very few bacteria were present in trials #2 and #3. For the reverse polarity, the latex substrates had more bacteria. 

These results show that the use of electrodes provides improvements compared with the control sample, where more than 90% of the surface was covered with rod shaped bacteria. Since bacteria are often encased in biofilm, a reduction in the amount of bacteria will also reduce the amount of biofilm. Based on this research, it can be seen that the presence of metals significantly reduces bacterial growth on latex. Furthermore, the application of voltage can essentially eliminate bacteria providing appropriate electrode combinations are used. 

## Figures and Tables

**Table 1:** Summary of Weekly Sample Appearance for Three Trials during Testing.

Sample	Week 1	Week 2	Week 3	Week 4
0 Control: Latex with nylon Mounting	Hydrophobic, Whitish-yellow discoloration near nylon bolt.	90% discolored and wetted on front and 25% on back with small protuberances.	100% wetted. 30% Coverage of small protuberances on front & some on back.	Similar to Week 3.
1 Latex with silver	Hydrophobic, No discoloration near bolt head.	50% discoloration on front with a few protuberances and 60% discoloration on back.	Hydrophilic, 60% Medium-sized bumps on front & 30% on back.	80% medium sized protuberances (bumps) on front and 80% on back.
2 Latex with stainless steel	Hydrophobic, discoloration near bolt head.	30% discoloration near metal on front and 15% discoloration on back.	Front hydrophilic. 80% large bumps on front & 20% small on back.	Similar to Week 3. White–yellow and grey discoloration on back.
3 Latex with brass	Hydrophobic with no discoloration near bolt head.	50% discolor on front near electrode & 50% discoloration on back.	Hydrophilic, 90% large bumps on front & 30% small ones on back.	Similar to Week 3. White–yellow and grey discoloration on back.
4 Latex with stainless steel–brass electrodes	Hydrophobic, discolor near stainless steel electrode & 50% discoloration on back.	60% discoloration on front near electrodes. 75% discoloration on back.	Hydrophilic with 50% small protuberances on front.	Similar to Week 3. White–yellow and grey discoloration on back and tip.
5 Latex with stainless steel–stainless steel electrodes and voltage applied	Hydrophobic, clear between electrodes, & 80% discolor on back.	50% discoloration on front, mostly around electrodes and 95% discoloration on back.	Hydrophilic with 80% small protuberances on front.	Similar to Week 3. White–yellow and grey discoloration on back.
6 Latex with brass–brass electrodes and voltage applied	Hydrophobic, clear between electrodes, & 40% discoloration on back.	40% discoloration on front and 80% discoloration on back.	Hydrophilic with 30% large protuberances on front and 10% small protuberances on back.	Similar to Week 3. Some additional discoloration on front.
7 Latex with brass positive electrode and stainless steel negative electrode	Hydrophobic. Discolor between electrodes & near brass electrode & 50% discoloration on back.	80% discoloration on front, particularly around brass electrode. 85% discoloration on back.	Hydrophilic with a few small protuberances on back.	Similar to week 3. Some discoloration on front.
8 Latex with brass negative electrode and stainless steel positive electrode	Hydrophobic, equal discolor near & between electrodes. 70% discoloration on back and near the brass.	85% discoloration on front and 95% discoloration on back.	Hydrophilic with a few protuberances.	20% small protuberances on front with some discoloration on front and back between electrodes.
